# Local Repair of a Secondary Aortoenteric Fistula in an Unstable Patient in a Resource-Poor Setting: A Case Report and Literature Review

**DOI:** 10.7759/cureus.14291

**Published:** 2021-04-04

**Authors:** Shariful Islam, Malini Ramnarine, Patrick Harnarayan, Anthony Maughn, Vijay Naraynsingh

**Affiliations:** 1 Department of General Surgery, San Fernando General Hospital, San Fernando, TTO; 2 Department of Clinical Surgical Sciences, University of the West Indies, St. Augustine, TTO; 3 Surgery, Medical Associates Hospital, St. Joseph, TTO

**Keywords:** aorto-enetric fistula, local repair, endovascular technique, outcomes, extra-anatomic bypass

## Abstract

The presentation of a massive upper gastrointestinal bleed (UGIB) due to an aortoenteric fistula (AEF) is a rare occurrence. A high index of suspicion is required to rapidly make the diagnosis and execute prompt surgical management. Despite the many surgical options described, the survival rate continues to be low. Conventional surgical management is associated with a high morbidity and mortality. However, in emergencies, patients are unsuitable for major vascular surgery and may benefit from the less invasive staged procedure. This is a case report of a secondary aortoenteric fistula (SAEF) presenting as a massive UGIB, two years after an abdominal aortic aneurysm repair using a Dacron graft. Due to a lack of endovascular service in our setting, we proceeded with an upper gastrointestinal endoscopy followed by exploratory laparotomy. A damage control approach was chosen for our patient, i.e., local repair of the graft and aorta, as our patient was on double inotropes on the table. The patient died within 24 hours as a result of massive blood volume loss.

## Introduction

As the evolution of vascular surgery occurred, so did the complications associated with each procedure. Secondary aortoenteric fistula (SAEF) is a rare but life-threatening postoperative complication of open repair of abdominal aortic aneurysms (AAA) [1]. The incidence of SAEF is 0.6% to 2%, and in 80% of the cases, it is usually associated with the 3rd or 4th parts of the duodenum [2]. Despite the improvement in the timing of diagnosis primarily due to the availability of newer imaging modalities, it is still associated with a high mortality rate, in the region of 50% [2,3]. The management of SAEF is still not well defined, as evidenced by the fact that a wide range of treatment options are still available, including both open or endovascular methods. However, a multidisciplinary approach is of great importance for a successful outcome.

The open procedures involve in situ aortic reconstructions, graft excision, extra-anatomic arterial bypass, and intestinal repair. These, however, are associated with higher mortality, especially in an unstable patient with multiple comorbidities [4,5]. Endovascular treatment such as balloon occlusion of the aorta, embolization, stent-graft repair has emerged as an alternative method over the last decade [3,6,7]; however, recent evidence suggests that it should be considered as a ‘bridging’ procedure because of high local recurrence rates [8].

Herein, we report the case of a 72-year-old patient who presented with a massive upper and lower gastrointestinal hemorrhage two years after an open elective repair of a AAA. Although rare, the likelihood of a SAEF was considered, but his instability precluded transfer to another facility, and he was treated by local repair of the graft along with gastrointestinal excision and closure.

## Case presentation

A 72-year-old male was presented to our Accident and Emergency Department with multiple episodes of per rectal bleeding and three episodes of hematemesis over the 12-hour period. He was a chronic smoker of greater than 50 packs per year and a chronic alcohol abuser. Surgical history revealed that he had abdominal aortic aneurysm repair using a graft (Dacron) prosthesis two years prior. On examination, he was slightly confused; his mucus membranes were dry and pale. His pulse was 128 beats per minute, and his blood pressure (BP) 76/47 mmHg. Abdominal examination revealed a reducible incisional hernia, and digital rectal examination revealed maroon color blood on the glove. All distal pulses were palpable. A diagnosis of massive upper gastrointestinal bleed (UGIB) was made. Resuscitation was initiated immediately, and the patient was referred to General Surgery and Gastroenterology.

**Figure 1:**
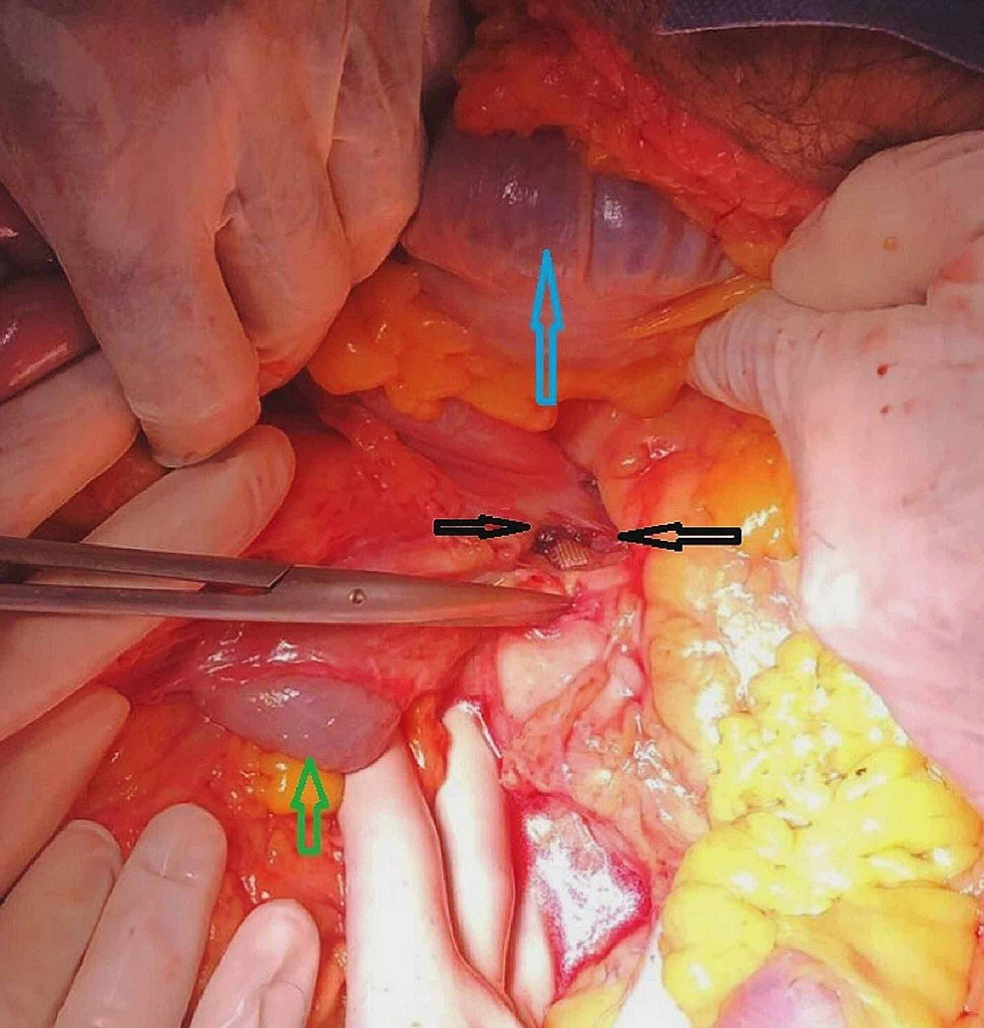
Intraoperative picture showing aortoenteric fistula (black arrow), blood in the transverse colon (blue arrow), and blood in the small intestine (green arrow)

A nasogastric tube (NGT) was carefully inserted with blood noted in the lumen of the tube. After the first liter of crystalloid infusion, the patient’s BP became 104/62 mmHg, but his pulse rate was still over 120 beats per minute. The hemoglobin level on admission was 8.9 gm/dl. As the patient was being prepared for a CT scan, 300 ml of blood drained from the NGT, and the BP started falling despite fluid resuscitation. The massive blood transfusion protocol was initiated. The gastroenterologist, anesthetist, and vascular surgeon were informed, and the patient was immediately taken to the operating theatre. Under general anesthesia with endotracheal tube intubation, the gastroenterologist performed upper gastrointestinal endoscopy, which failed to identify the source of bleeding. The abdomen was then cleaned and draped. A midline laparotomy was immediately performed. An incision was made over the pylorus of the stomach and extended into the 1st part of the duodenum. The source of bleeding was assessed; however, blood was found to be pouring from the distal duodenum. Supra-celiac clamping of the aorta was performed to gain proximal control, and distal control was achieved by bilateral clamping the common iliac artery. The entire duodenum was mobilized. It was noted that the proximal jejunum (approximately 10 cm from the DJ (duodenojejunal) junction) was very adherent to the aorta (Figure 1).

**Figure 2:**
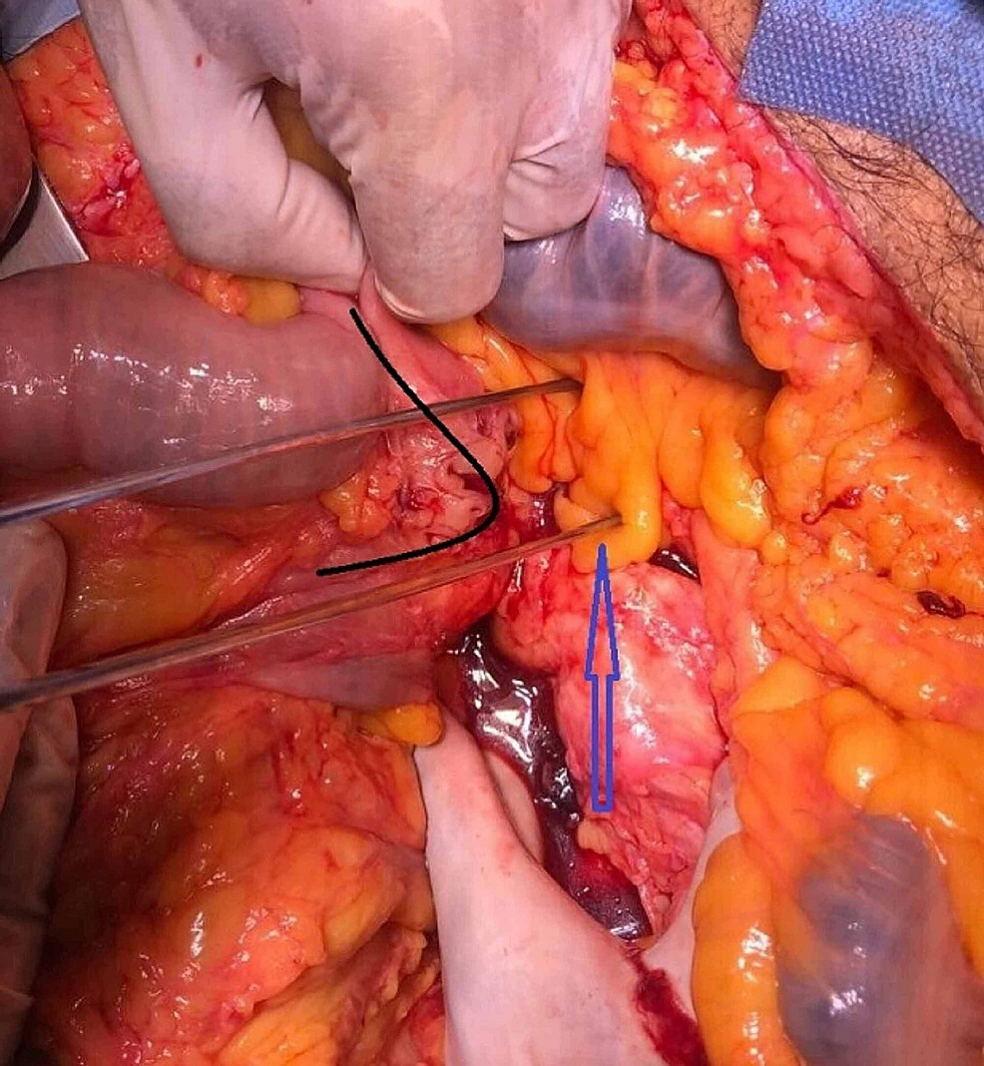
Intraoperative picture showing the aortoenteric fistula – the loop involved is the proximal jejunum (black arrow), the aorta (blue arrow)

On further exploration, a fistula was noted between the proximal end of the anastomosis of the previous graft (Dacron) repair and the proximal jejunum because of the graft eroding into the jejunum (Figure 2).

**Figure 3:**
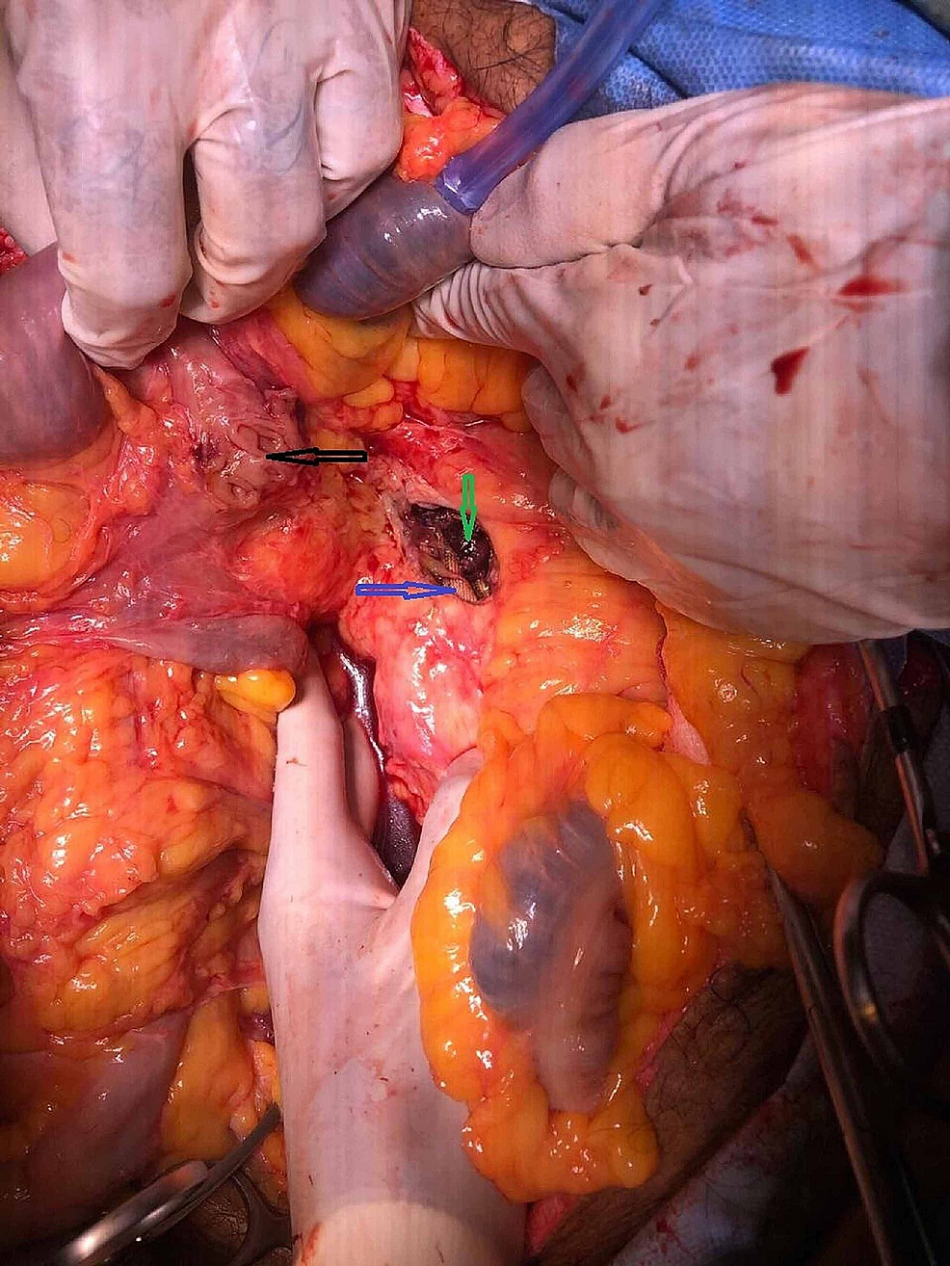
Intraoperative picture demonstrating the proximal jejunum dissected off the aorta: opening in the proximal jejunum (black arrow), exposed graft at the proximal aortic anastomosis (blue arrow), clots within the aorta (green arrow)

The proximal jejunum was completed dissected off the aorta revealing the exposed graft at the proximal anastomosis and thrombus within the lumen, as shown in Figure 3.

Unfortunately, our patient had lost significant blood volume at this stage, and he was on double inotropes; a damage control approach was chosen for his management. The edges of the fistula opening of the jejunum were refreshed and repaired. The edges of the aorta and the graft were debrided, and the graft was re-anastomosed with the aorta. The excised edge of the graft was sent for culture and sensitivity. The pyloromyotomy opening was closed using the Heineke-Mikulicz pyloroplasty technique. Two large drains were left in situ, and the abdomen was closed. The patient was transferred to the intensive care unit post-surgery. He received multiple blood and blood products but unfortunately demised within 24 hours due to massive blood loss.

## Discussion

The abnormal communication between the aorta and the gastrointestinal tract is termed an aortoenteric fistula (AEF) which can further be classified into primary AEF (PAEF) and secondary AEF (SAEF). PAEF was first described by Sir Astley Cooper in 1829 [3], and it carries a prevalence of 0.04%-0.07% [6]. The most common cause is an aneurysm of the aorta and less common foreign body, radiation treatment, tumors, and infections such as tuberculosis and syphilis.

SAEF was first described by Brock in 1953 [6] with a slightly greater prevalence of 0.77%-1.6% [9] than PAEF. The most common cause of SAEF is the erosion of the graft material into the third part of the duodenum at the proximal anastomotic site. In our case, the site of AEF was 10 cm distal to the DJ junction. 

There is much controversy regarding the ideal treatment for SAEF, with no established guidelines for surgeons to follow [10,11]. The goal of treatment for SAEF includes, most importantly, early vascular control, debridement up to healthy tissues (aorta and intestines), removal of infected graft, revascularization, and restoring continuity of the gastrointestinal tract [12].

In patients who are unstable, have multiple comorbidities, or are poor candidates for major surgery, endovascular options such as balloon occlusion of the aorta, embolization, stent-graft repair, plus repair and injection of fibrin sealant into the fistula can be utilized in the management of SAEF in an attempt to decrease the morbidity and mortality [13,14].

Endovascular interventions in SAEF correlate with better short-term results when compared with open repairs (33.9% vs. 7.1%, p<0.001). However, these survival rates decreased to 51% and 40%, with higher rates of sepsis of 42% vs. 19% respectively at two years follow up by Kakkos et al. [15]. The higher rates of recurrent bleeding and sepsis are attributed to the limitation that endovascular therapy does not include repair of the intestinal defect and hence cannot be used as the definitive treatment of SAEF but rather as a bridging therapy to open repair [15].

Ongoing blood loss and shock at presentation are major risk factors for an unfavorable outcome. The advent of a promising technology such as resuscitative endovascular balloon occlusion of the aorta (REBOA) is showing new hope amongst the trauma surgeons [16] for the management of traumatic non-compressible torso hemorrhage patients [17-19].

A recent meta-analysis and systematic review by Manzano Nunez et al. compared the mortality among the REBOA to resuscitative thoracotomy in 1276 non-compressible traumatic torso hemorrhage patients. A positive effect on mortality was noted among the REBOA patients compared to those who underwent resuscitative thoracotomy (RT) [20].

Early involvement of senior surgeons with vascular experience or interventional radiologists is paramount for a successful outcome to be achieved. In this case, the initial suspicion of a duodenal ulcer or oesophageal varix rather than the more uncommon aortoenteric fistula as the etiology of the gastrointestinal hemorrhage delayed the diagnosis. Since endoscopy is routinely done in all cases of severe upper gastrointestinal hemorrhage, this was performed, but the exact site of origin of the hemorrhage could not be accurately ascertained. It is possible that the procedural time spent during endoscopy and the initial surgical incision to inspect the contents of the gastric region (pyloromyotomy) may have led to an unnecessary prolongation in the overall operating time. The excess blood loss could have also have been reduced by early deployment of an intra-aortic balloon. Unfortunately, this service is not available at our hospital, which forces us to proceed with the only available option at hand, aortic cross-clamping and local repair of the graft and the intestine.

## Conclusions

The diagnosis of SAEF should be considered in all patients presenting with upper GI bleeding with a history of AAA repair. The management of SAEF is challenging and necessitates a prompt multidisciplinary approach. The involvement of the specialist surgeons at the early stage of management is necessary for a successful surgical outcome. Any unnecessary steps should be avoided, as time spent during these procedures could have an adverse effect on survival. Unwanted blood loss can be reduced by prior occlusion of the aorta by endovascular means via an intra-aortic balloon. However, in a resource-poor setting like ours, we think an early cross-clamping of the aorta can be the only option prior to the pyloromyotomy to prevent unwanted blood loss. Similarly, local repair of an aortoenteric fistula can be used as a "bridge therapy" in unstable patients. 
